# Physical literacy as a determinant of physical activity level among late adolescents

**DOI:** 10.1371/journal.pone.0285032

**Published:** 2023-04-28

**Authors:** Özgül Öztürk, Onur Aydoğdu, Seval Kutlutürk Yıkılmaz, Özlem Feyzioğlu, Pelin Pişirici

**Affiliations:** 1 Department of Physiotherapy and Rehabilitation, Acıbadem Mehmet Ali Aydınlar University, Istanbul, Turkey; 2 Department of Physiotherapy and Rehabilitation, Marmara University of Health Sciences, Istanbul, Türkiye; 3 Department of Physiotherapy and Rehabilitation, University of Health Sciences, Istanbul, Türkiye; 4 Department of Physiotherapy and Rehabilitation, Bahçeşehir University, Istanbul, Türkiye; Nnamdi Azikiwe University, NIGERIA

## Abstract

**Background/Objectives:**

This study aimed to investigate the level of physical literacy among late adolescents according to the current physical activity level and to examine the relationship between current physical activity, barriers to engaging in physical activity, and enjoyment of physical activity and physical literacy.

**Methods:**

A total of 568 university students (405 women) aged from 18 to 20 were involved in this study. The physical literacy, physical activity level, enjoyment from activity, and barriers to physical activity were assessed with the Perceived Physical Literacy Instrument (PPLI), the International Physical Activity Questionnaire–Short Form (IPAQ–SF), Physical Activity Enjoyment Scale (PACES), and the Physical Activity Barriers Questionnaire (PABQ), respectively. Multinomial and binary logistic regression analyses were employed to explore the association between physical literacy and physical activity level.

**Results:**

Highly physically active adolescents had better scores on the PPLI, PACES, and PABQ than moderately active and inactive participants. The PPLI total score was significantly moderately correlated with PACES total, positive, and negative scores and the PABQ score. There were significant poor correlations between the IPAQ-MET value and the PPLI scores. Adjusted logistic regression analysis revealed the PPLI total score and the PACES positive sub-scale scores, and gender (men) were associated with being highly active relative to moderately active.

**Conclusions:**

The findings highlight the evidence that physical literacy, gender, and enjoyment from activity can be determinants of high or moderate physical activity levels. Therefore, improving physical literacy among late adolescence may be key to achieving increased physical activity level.

## 1. Introduction

Physical activity is defined as any form of body movement produced by skeletal muscle contractions that leads to energy expenditure [1]. Increased participation in physical activity programs is integral to prevent adverse health outcomes across the life course [2]. Also, physical activity has benefits for mental health and well-being [3,4]. Despite this, only 30.3% of the university students meet physical activity recommendations by performing 30 minutes of moderate to vigorous physical activity for five days in a week [5]. Positive adolescent well-being enhances overall health and decreases risky health behaviors during adulthood [6], thus promoting physical activity in the young population would bring significant benefits for the rest of life.

Physical literacy is described as the competence, confidence, and knowledge required to be physically active throughout life [7]. Physical literacy, as a multidimensional concept, has physical, social, cognitive, and affective domains which have been positioned as a health and disease determinant [8]. High levels of physical literacy may be associated with better health outcomes in terms of cardiovascular fitness, muscle strength, motor skills, and obesity status [9,10]. Motivation, self-efficacy, and knowledge as well as understanding the potential benefits of physical activity within a physical literacy concept, may increase physical activity levels in late adolescence [11]. A person with high physical literacy can be more physically active, engage in various physical activities, and have a higher level of physical fitness.

Achieving the optimal level of physical literacy in early life may affect physical activity participation and may prevent chronic diseases in adulthood [8]. Besides physical skills, physical literacy is also about developing the attitudes and motivation that can influence physical activity participation [11]. Especially, individuals in adolescence period should be physically literate to realize the importance of being physically active and increase their level of physical activity [8]. Late adolescents, typically defined as individuals between the ages of 16 and 20, are at a crucial stage of physical development and can benefit greatly from being physically literate. Also, university students at this age group may face barriers to engage physical activity programs considering the transition from late adolescence into adulthood as a major step that changes priorities and decreases physical activity participation [12]. Lack of time, lack of motivation, inaccessibility to sports facilities, and lack of social support have been described as barriers to physical activity participation [13]. Positive lifestyle behaviors that will be settled in this time frame may foster lifelong physical activity habits and help to achieve the main goal of having a healthy active life. World Health Organization (WHO) emphasized the importance of physical literacy by mentioning the role of this concept in a physical activity action plan at the national level [14].

The relationship between physical activity and physical literacy has been reported in adolescents by showing gender, socioeconomic status, and grade level as individual factors in this issue [15]. However, to our knowledge, limited evidence is available related to the relationship between physical literacy and physical activity level by considering other associated factors in late adolescents.

Therefore our aim was to investigate the level of physical literacy among late adolescents according to the current physical activity level and to examine the relationship between current physical activity, barriers to engaging in physical activity, and enjoyment of the physical activity and physical literacy.

## 2. Materials and methods

### 2.1.Participants

This cross-sectional study was conducted with first and second-degree physiotherapy and rehabilitation students in public and private universities who can be classified as late adolescents. The participants were included if they were 18 to 20 years old and volunteer to participate. Participants were excluded if they had an orthopedic or neurological problem that may affect physical activity participation. The survey was performed using the Google Forms platform and a link including the questions was sent to the participants. The study protocol was approved by Acıbadem Mehmet Ali Aydınlar University ethics committee (ATADEK 2022/16) and written informed consent was obtained from the participants. Demographic variables were collected (age, gender, cigarette/alcohol usage, and information about sports habits).

### 2.2.Assessments

Self-perceived physical literacy of adolescents was assessed with the Perceived Physical Literacy Instrument (PPLI) which has three subscales: “knowledge and understanding”, “self-expression and communication with others”, and “self-confidence”. The PPLI consists of 9 items scored from 1 (strongly disagree) to 5 (strongly agree), the total score ranges from 5 to 45, and higher scores indicate a high level of physical literacy [16,17].

Physical activity level was determined using the International Physical Activity Questionnaire–Short Form (IPAQ–SF), which asks the number of days and average time spent during vigorous and moderate activities and walking [18]. Participants were classified as inactive, moderately active, and highly active and the metabolic equivalent of task (MET) values were calculated. To determine the MET values per week, the MET values assigned to specific activity (walking = 3.3 METs, moderate activity = 4 METs, and vigorous activity = 8 METs) were multiplied with the duration of the activity in minutes, and the number of the days that the activity [19]. The IPAQ-SF displayed significant a moderate correlation with accelerometer data in university students [20].

Physical Activity Enjoyment Scale (PACES) comprises 15 items on a 5-point Likert-type scale (1 = "totally disagree"; 5 = "totally agree"). This scale consists of two subscales: the first subscale includes items indicating positive thoughts about exercising and being active (8 items in total) and the second subscale includes negative items such as disliking or not having fun with physical activity (7 items in total). The maximum score is 75 and the minimum score is 15, the higher score indicates higher enjoyment of activity [21].

Barriers to physical activity were assessed with the Physical Activity Barriers Questionnaire which includes 24 items on a 5-point Likert-type scale (1 = "strongly disagree"; 7 = "strongly agree). A higher score given to each item indicates a higher likelihood to have a barrier to physical activity [22].

### 2.3.Statistical analysis

Data were analyzed using SPSS 22.0 (IBM Corporation, Armonk, New York) and GraphPad Prism 8 (GraphPad Software Inc., CA). Grubb’s test was used to detect outliers and whether outliers were present at an alpha of 0.05. In the case of finding an outlier, that value was removed from the dataset. The normal distribution of the data was examined using visual (histogram and probability plots) and analytical (Shapiro–Wilk Test) methods. The descriptive statistics are presented as median and interquartile range or mean and standard deviation for the quantitative variables and frequency and percentage values for the qualitative variables. Gender differences were investigated using Mann-Whitney-U or Independent-t-test for continuous variables and the chi-squared test for categorical variables.

Two different two-way multivariate analysis of variance (MANOVA) was performed with two factors (physical activity level and gender) to assess the differences between PPLI and PACES scores. Also, a two-way ANOVA with the same factors was conducted to assess differences in the Physical Activity Barriers Questionnaire score. Partial eta square (η^2^) was calculated to interpret the effect sizes using the following criteria: below 0.04 as no effect, between 0.04 to 0.25 as a minimum effect, between 0.25 to 0.64 as a moderate effect, and above 0.64 as a strong effect. Tukey’s test was conducted for post-hoc analysis.

Pearson correlation analysis was performed to reveal possible correlations between physical literacy, MET value, level of enjoyment, and barriers to physical activity.

To explore the association between physical activity level, physical literacy, enjoyment from physical activity, and barriers to physical activity, two logistic regression models were undertaken. A multinomial logistic regression model was utilized by considering the physically inactive group as the reference category. Candidate variables were gender (the female was the reference group), BMI, smoking and alcohol usage, PPLI scores, PACES scores, and barriers to physical activity. Unadjusted and adjusted (BMI, smoking, and alcohol usage) odds ratios (OR) with 95% confidence intervals were reported. The goodness of fit of the constructed model was checked using the deviance statistic and the pseudo R (Nagelkerke). The statistical significance was set at the 0.05 level.

## 3. Results

A total of 568 students (405 women) participated in this study. Missing values were imputed by applying multiple imputation methods. Of the participants, 9% of them were physically inactive, 54.2% of them were moderately active, and 36.8% of them were highly active according to the IPAQ-SF. Also, self-reported physical activity levels displayed similar results as 6.5%, 58.3, and 35.2% for being physically inactive, moderately active, and highly active, respectively.

According to the results of Grubb’s test, data related to one participant were removed for PPLI total, knowledge, and self-expression scores and PACES scores (positive and negative subscale) because of being outliers.

Because of the higher contribution of women in the present study, we applied a subgroup analysis and the results indicated that the BMI, smoking status, sports participate level, both self-reported and the IPAQ-SF-determined physical activity level, and the PPLI scores (total, self-confidence, self-expression, and knowledge) were different in favor of men. Also, the Physical Activity Barriers Questionnaire score was lower in men than in women (Table 1).

**Table 1:** The characteristics of participants stratified by gender.

Variables	Total (n = 568)	Women (n = 405)	Men (n = 163)	p value
BMI, kg/m^2^, mean (SD)	22.21 (3.69)	21.52 (3.50)	24.10 (3.50)	0.001*
Currentsmokers,yes, n (%)	124 (21.8)	71 (17.5)	53 (32.5)	0.001*
Currentalcoholusers, yes, n (%)	159 (28.0)	105 (25.9)	54 (33.1)	0.098
Sportparticipatinglevel (professional), n (%)	67 (11.8)	29 (7.2)	38 (23.3)	0.001*
Self-reportedphysicalactivitylevel, n (%)				0.001*
Physicallyinactive	37 (6.5)	33 (8.1)	4 (2.4)	
Moderatelyactive	331 (58.3)	259 (64.0)	72 (44.2)	
Highlyactive	200 (35.2)	113 (27.9)	87 (53.4)	
IPAQ-SF				0.001*
Physicallyinactive	51 (9.0)	38 (9.4)	13 (8.0)	
Moderatelyactive	308 (54.2)	254 (62.7)	54 (33.1)	
Highlyactive	209 (36.8)	113 (27.9)	96 (58.9)	
IPAQ,-SF, MET value, median (IQR)	1945 (1173–3547)	1695 (1074–2746)	3192 (1398–5346)	0.001*
PPPLI total score, mean (SD)	35.09 (4.66)	34.57 (4.36)	36.38 (5.14)	0.001*
Self-confidence	10.98 (2.32)	10.80 (1.96)	11.43 (2.38)	0.003*
Self-expression	11.11 (1.99)	10.85 (1.96)	11.76 (1.94)	0.001*
Knowledge	13.01 (1.62)	12.91 (1.54)	13.28 (1.78)	0.014*
PACES total score,mean (SD)	61.56 (7.87)	61.51 (7.51)	61.66 (8.75)	0.837
Positive	32.22 (4.61)	32.09 (4.43)	32.55 (5.02)	0.286
Negative	29.45 (3.90)	29.42 (3.68)	29.53 (4.41)	0.787
PhysicalActivity BarriersQuestionnaire, mean (SD)	51.74 (13.25)	53.40 (12.08)	47.63 (15.03)	0.001*

Abbreviations: BMI, Body Mass Index; PPLI, PerceivedPhysicalLiteracyInstrument; PACES, Physical Activity Enjoyment Scale; IPAQ-SF; International Physical Activity Questionnaire Short Form; MET; Metabolic Equivalent.

* Statistical significance (p < 0.05).

For comparison of physical literacy according to the physical activity level and gender, the two-way MANOVA showed that the physical activity level had a significant effect on PPLI total, self-confidence, self-expression, and knowledge sub-scores (p = 0.001, η^2^ = 0.049), however, no effect was found for gender on PPLI scores (p = 0.356, η^2^ = 0.006). The physical activity level and gender had no interaction (Pillai’s Trace = 0.020, p = 0.074, η^2^ = 0.01). Post-hoc analysis showed that significant differences were found for the PPLI total score and self-expression sub-scores between highly active than moderately active (p = 0.001) and inactive individuals (p = 0.001). Also, the PPLI total score (p = 0.003) and self-expression sub-scores (p = 0.001) were different between moderately active and inactive participants. The PPLI self-confidence and knowledge sub-scores were different between highly active compared to moderately active (p = 0.001) and inactive (p = 0.001) individuals. No differences were found between moderately active and inactive participants (p > 0.05) (Table 2).

**Table 2:** Comparison of the BMI, physical literacy, enjoyment from physical activity, and, barriers to physical activity based on physical activity level.

Variables	Physically inactive (n = 51)	Moderately active (n = 308)	Highly active (n = 209)	P value
BMI, kg/m^2^, mean (SD)	22.41 (3.95)	21.95 (3.45)	22.68 (3.92)	0.065
PPPLI total score,	32.19 (5.24)	34.39 (4.47)	36.81 (4.17)	0.001*
Self-confidence	10.01 (2.55)	10.71 (2.32)	11.62 (2.11)	0.001*
Self-expression	9.90 (2.27)	10.92 (1.93)	11.67 (1.84)	0.001*
Knowledge	12.27 (1.87)	12.80 (1.54)	13.51 (1.53)	0.001*
PACES total score,	58.84 (8.60)	60.57 (7.20)	63.67 (8.19)	0.001*
Positive	30.60 (5.03)	31.60 (4.23)	33.53 (4.74)	0.001*
Negative	28.23 (4.37)	29.12 (3.53)	30.24 (4.16)	0.001*
Physical Activity BarriersQuestionnaire	55.68 (12.43)	52.96 (11.76)	48.96 (14.95)	0.006*

Abbreviations: BMI, Body Mass Index; PPLI, PerceivedPhysicalLiteracyInstrument; PACES, Physical Activity Enjoyment Scale; IPAQ-SF; International Physical Activity Questionnaire Short Form; MET; Metabolic Equivalent.

* Statistical significance (p < 0.05).

The two-way MANOVA revealed that the physical activity level had a significant effect on PACES total, positive, and negative scores (p = 0.001, η^2^ = 0.024), while no effect was found for gender on PACES scores (p = 0.512, η^2^ = 0.002). There was no interaction between the physical activity level and gender (Pillai’s Trace = 0.002, p = 0.895). Post-hoc analysis showed that PACES total, positive, and negative scores were higher in highly active people compared to moderately active (p = 0.001, p = 0.002, p = 0.001 for PACES total, positive, and negative scores, respectively) and inactive individuals (p = 0.001, p = 0.003, p = 0.001 for PACES total, positive, and negative scores, respectively) (Table 2).

Physical Activity Barriers Questionnaire differed among the physical activity level according to the two-way ANOVA (F = 5.21, p = 0.006, η^2^ = 0.01) and gender (F = 5.50, p = 0.019, η^2^ = 0.01), however, there was no interaction between gender and physical activity level (F = 0.58, p = 0.558). Post-hoc analysis showed that highly active people had fewer barriers compared to moderately active (p = 0.002) and inactive individuals (p = 0.003) (Table 2).

Pearson correlation analysis showed that the PPLI total score was significantly moderately correlated with PACES total, positive, and negative scores. Also, a significant negative moderate correlation was found between the PPLI and Physical Activity Barriers Questionnaire score. There were significant positive poor correlations between the IPAQ-MET value and the PPLI total and sub-scores, the PACES total and sub-scores. A significant negative poor correlation was found between the IPAQ-MET value and barriers score (Table 3).

**Table 3:** Correlation between physical literacy, MET value, level of enjoyment, and barriers to physical activity.

Variable	Correlation Coefficient (r)	p Value
*PPLI total score*		
PACES total score	0.527	0.001*
PACES positive subscale	0.511	0.001*
PACES negative subscale	0.448	0.001*
Physical Activity Barriers Questionnaire	-0.483	0.001*
*IPAQ-SF MET value*		
PPLI total score	0.330	0.001*
PPLI self-confidence	0.236	0.001*
PPLI self-expression	0.242	0.001*
PPLI knowledge	0.300	0.001*
PACES total score	0.252	0.001*
PACES positive subscale	0.248	0.001*
PACES negative subscale	0.209	0.001*
Physical Activity Barriers Questionnaire	-0.260	0.001*

Abbreviations: PPLI, PerceivedPhysicalLiteracyInstrument; PACES, Physical Activity Enjoyment Scale; IPAQ-SF; International Physical Activity Questionnaire Short Form; MET; Metabolic Equivalent.

* Statistical significance (p < 0.05).

A backward elimination strategy was applied for multinomial analysis and variables that did not exhibit a significant association with physical activity level at a p<0.20 were removed from the analysis. The PPLI total score, gender (reference category women), and the PACES positive sub-scale score remained significant in the model. Multiple multinomial logistic regression in unadjusted analysis clarified the impact of the PPLI total score (Odds Ratio (OR) = 1.11, 95% confidence interval (CI)1.03–1.19) on moderately active status relative to physically inactive. The PPLI total score (OR = 1.20, 95% CI 1.11–1.29) and gender (OR = 0.45, 95% CI 0.22–0.95) were associated with being highly active relative to physically inactive. In adjusted analysis, only the PPLI total score had an impact on being moderately active (OR = 1.10, 95% CI 1.03–1.19) and highly active relative (OR = 1.20, 95% CI 1.11–1.31) to being physically inactive (Table 4).

**Table 4:** Multinomial logistic regression analyses for moderately active or highly active participants relative to inactive.

Measures	Moderately active relative to inactive			Highly active relative to inactive			Moderately active relative to inactive			Highly active relative to inactive		
	Unadjusted						Adjusted^a^					
	OR	95% CI	p value	OR	95% CI	p value	OR	95% CI	p value	OR	95% CI	p value
PPLI total score	1.11	1.03–1.19	0.003*	1.20	1.11–1.29	0.001*	1.10	1.03–1.19	0.005*	1.20	1.11–1.31	0.001*
Gender (reference category women)	1.73	0.84–3.53	0.131	0.45	0.22–0.95	0.037*	1.66	0.78–3.55	0.188	0.50	0.23–1.08	0.079
PACES positive sub-scale	0.98	0.91–1.06	0.712	1.05	0.97–1.13	0.213	0.98	0.91–1.05	0.616	1.04	0.96–1.13	0.271

Abbreviations: OR, oddsratio; CI, confidentialintervals; PPLI, PerceivedPhysicalLiteracyInstrument; PACES, Physical Activity Enjoyment Scale.

^a^Adjustedfor body massindex, smoking, alcoholusage.

* Statistical significance (p < 0.05).

Binary logistic regression analysis was performed to identify the associated factors for being highly active relative to moderately active. After adjusting for potential confounding factors (BMI, smoking, and alcohol usage), the PPLI total score (OR = 1.07, 95% CI 1.02–1.13) and the PACES positive (OR = 1.06, 95% CI 1.01–1.12) sub-scale scores remained significant in the model by showing positive association. Gender (men) was negatively associated with being highly active relative to moderately active (OR = 0.14, 95% CI 0.03–0.32) (Table 5).

**Table 5:** Logistic regression analysis for highly active relative to moderately active individuals.

	Unadjusted			Adjusted ^a^		
	OR	95% CI	p value	OR	95% CI	p value
Gender (reference category women)	0.30	0.19–0.48	0.001*	0.14	0.03–0.32	0.001*
PPLI total score	1.08	1.02–1.14	0.003*	1.07	1.02–1.13	0.005*
PACES positive sub-scale	1.06	1.01–1.12	0.011*	1.06	1.01–1.12	0.010*

Abbreviations: OR, oddsratio; CI, confidentialintervals; PPLI, PerceivedPhysicalLiteracyInstrument; PACES,Physical Activity Enjoyment Scale.

^a^Adjustedfor body massindex, smoking, alcoholusage.

* Statistical significance (p < 0.05).

## 4. Discussion

The main finding of this cross-sectional study is that the level of physical literacy differs among individuals with high, moderate, and low physical activity levels. Also, highly active participants had more enjoyment from the activity and reported lower barriers to physical activity compared to moderately active and physically inactive individuals. Moreover, physical literacy, enjoyment from physical activity, and gender can be served as determinants of being highly or moderately physically active. In addition to this, physical literacy is positively correlated with enjoyment from the activity and MET value, and negatively correlated with barriers to being physically active.

A physically active lifestyle during late adolescence can have a positive impact on physical activity participation and health outcomes in adulthood, making it a critical period for promoting physical activity and establishing healthy habits [23,24].Thus, there is a need for increased efforts to promote physical activity in late adolescence to provide a range of benefits such as the reduced risk of chronic diseases, improved physical fitness, and increased quality of life [25]. However, as individuals age, there is an increase in studying time at school which may lead to a decrease in time spent on daily physical activities and a consequent increase in sedentary time [26]. Also, low levels of physical activity cause deleterious effects on the psychological status of university students [27]. In our study, 36.8% of the participants were classified as highly active, 54.2% were moderately active, and only 9.0% of them were physically inactive according to the IPAQ-SF. In addition to this, self-reported physical activity levels displayed similar results to the IPAQ-SF (6.5% as inactive and 35.2% as highly active). Since physical inactivity was reported as 21.9% to 80.6% depending on the country, we compared our results with the Turkish population and in that study, 24.6% of Turkish students were categorized as inactive, 40.8% as moderately active, and 34.6% as highly active [28]. The discordance between our results with the above-mentioned study may be explained by the proportion of participants who were performing a regular sportive activity (11%) at a professional level which may increase the number of highly physically active people. Also, a majority of physiotherapy students (65%) engage in regular sportive activities because of the knowledge about the benefits of physical activity [29], which may be a possible reason for the lower proportion of physically inactive participants in the present study.

Physical literacy has been investigated in children and young adolescents reflecting 85% of inadequate physical literacy level [30]. Also, the possible relationship between physical literacy and physical activity is an area of interest in various populations. Choi et al. found a significant weak correlation between physical literacy and physical activity level in adolescence (r = 0.22) [15]. In line with this result, we found significant correlations between physical literacy components and MET value varied from 0.23 to 0.33. Also, our results indicated that highly active participants had an increased physical literacy level compared to moderately active or inactive people. Considering the sociodemographic characteristics, men were more physically literate than women, however, gender is not a game-changing factor for determining physical literacy status among the groups categorized by physical activity level. Promoting physical literacy and encouraging physical activity in this age group is vital to reach a more highly physically active adolescence.

Physical literacy encompasses not only physical skills and abilities but also confidence and enjoyment in physical activity [31]. Research has shown that enjoyment is strongly related to physical literacy [32] and also adolescence who feel enjoyment from physical activity are more likely to stick with it [33], leading to increased physical fitness and overall health. In line with this knowledge, highly active individuals reported more enjoyment from physical activity and physical literacy was correlated with enjoyment from physical activity. Therefore, physical literacy and enjoyment from physical activity can be presented as determinants for being moderately or highly physically active.

The transition from high school to university causes increased barriers to physical activity in terms of higher school workload and more time spent on social interaction rather than physical activity [34]. Individuals with low physical literacy may face additional barriers to physical activity, such as a lack of confidence or skill in physical activities and limited motivation [35]. In the present study, barriers to physical activity were moderately correlated with physical literacy. This highlights the importance of promoting physical literacy to overcome barriers to physical activity, prevent a typical decline in physical activity levels, and encourage physical activity participation.

This study is not without limitations. Firstly, we conducted a cross-sectional study, longitudinal or prospective studies are needed to define the causal relationship between physical activity level and physical literacy in late adolescence. Secondly, we used a self-reported tool to define the physical activity level which may lead to recall and reporting bias. Thirdly, our subjects comprise physiotherapy and rehabilitation students from four different universities, so caution should be taken to generalize our findings to all university students. Despite the above limitations, the present study first highlights the importance of association between physical literacy and physical activity level.

To conclude, our results indicated that physical literacy, enjoyment from the activity, and being male gender were related to physical activity levels among university students. Also, physical literacy was correlated with barriers to physical activity and enjoyment from activity. Thus, the close relationship between physical literacy, physical activity, and associated factors may lead the researchers to the importance of physical literacy as a promising way to promote physical activity in late adolescence.
